# Parathyroid identification by autofluorescence – preliminary report on five cases of surgery for primary hyperparathyroidism

**DOI:** 10.1186/s12893-019-0590-9

**Published:** 2019-08-28

**Authors:** Carlos Serra, Luís Silveira, António Canudo, Manuel C. Lemos

**Affiliations:** 1 Departamento de Cirurgia, Hospital dos SAMS, Lisbon, Portugal; 2 CICS – UBI, Health Sciences Research Centre, Universidade da Beira Interior, Covilhã, Portugal; 3 Faculdade de Ciências da Saúde, Universidade da Beira Interior, Covilhã, Portugal; 4 Departamento de Cirurgia, Hospital dos SAMS, Lisbon, Portugal

**Keywords:** Autofluorescence, Parathyroid, Surgery, Near infrared

## Abstract

**Background:**

Intra-operative identification of parathyroid glands is often a challenge for surgeons performing parathyroid or thyroid surgery. Parathyroid glands stimulated by near-infrared light emit autofluorescence, which allows their discrimination from all other tissues in the region, and this may be of value during thyroid and parathyroid surgery. In this study, we present the results of the utilization of a low-cost device developed for the identification of parathyroid glands in surgery for primary hyperparathyroidism.

**Case presentation:**

In 5 patients operated in our hospital with the diagnosis of primary hyperparathyroidism and non-concordant ultrasonography and Sestamibi scan, we used a 780 nm Light Emitting Diode (LED) to stimulate the cervical area. The resulting autofluorescence was visualized with night vision goggles with a 832 nm filter assembled.

In all the five patients, an easily distinguishable nodule was identified and excised, and confirmed as parathyroid adenoma by histological exam. Intra-operative PTH assay showed significant decrease compared with basal values, fulfilling the Miami Criteria for surgical success in use in our institution.

**Conclusion:**

The utilization of autofluorescence for intra-operative identification of parathyroid glands may have a clinical application in surgery for primary hyperparathyroidism, being of special utility when ultrasonography and Sestamibi Scan are non concordant.

## Background

Intra-operative identification of parathyroid glands is often a challenge for surgeons performing parathyroid or thyroid surgery. Their small size, shape and color makes them difficult to differentiate from other cervical tissues such as lymph nodes, fat and even small thyroid nodules [1, 2].

Since the beginning of parathyroid surgery, the identification of these glands has depended mainly on the surgeons’ visual acuity and experience. Almost all the available techniques that could help surgeons for this purpose are dependent of the injection of exogenous substances with associated toxicity risks [3, 4].

In 2011, Paras et al. [5] reported for the first time the autofluorescence properties of parathyroid glands when stimulated with a laser beam of 795 nm, which allowed their differentiation from other tissues in the area [5]. Subsequently, other authors confirmed this property, with applications in thyroid and parathyroid surgery [6–9].

The non ionizing nature of near infrared light (700–2500 nm) and its capacity of penetration in biological tissues makes it a promising tool for image-guided surgery [10, 11].

Current devices capable of detecting intraoperative autofluorescence are generally expensive. Therefore, the authors developed a low-cost device, assembled with easily obtained components, and report its use in a group of patients with primary hyperparathyroidism that had discordant imaging localization tests.

## Case presentation

This study was conducted from June to October 2018 in Hospital dos SAMS, Lisbon, Portugal after approval by the Institutional Ethics Committee.

Following the almost universally accepted practice for the surgery of primary hyperparathyroidism, in Hospital dos SAMS we favour the focused lateral approach, based on the pre-operative localization of the pathological parathyroid gland by two concordant tests (cervical ultrasonography and Sestamibi Scan), controlled by intraoperative PTH analysis using Miami criteria [12–14].

In case of non concordant tests, a bilateral cervical exploration is usually performed through a collar incision, with identification of four glands. If localization tests point to the same side of the neck, an unilateral exploration with identification of the two glands on that side could be undertaken.

Five patients (1 male, 4 females, median age 57 years, range 26–72 years) with clinical and laboratorial diagnosis of primary hyperparathyroidism, but with non-concordant localization tests, were referred for surgical treatment and were included in the study (Table 1). Two patients also had indication for homolateral thyroid lobectomy, which was performed in the same surgical time.

**Table 1:** Characteristics of patients

Patient	Sex	Age (years)	Diagnosis	Non Concordance
1	F	51	Primary Hyperparathyroidism	Sestamibi -Hyperfixation on PIII right Ultrasound – PIII left
2	F	72	Primary Hyperparathyroidism	Sestamibi - negative Ultrasound- PIII right
3	F	68	Primary Hyperparathyroidism Nodule on right lobe of thyroid	Sestamibi - negative Ultrasound– intrathyroid nodularity
4	F	69	Primary Hyperparathyroidism Nodule on right lobe of thyroid	Sestamibi- PIV left Ultrasound- PIV right
5	M	26	Primary Hyperparathyroidism	Sestamibi- PIII right Ultrasound - negative

Abbreviations: *PIII* inferior parathyroid, *PIV* superior parathyroid

Patients were submitted to a cervical exploration through a collar incision. After opening the median raphe, we proceeded with visual inspection of the area, starting by the side where Sestamibi Scan showed hyperfixation of the radiotracer, or by the side where ultrasonography showed a suspected nodule if Sestamibi scan was negative.

After visual inspection and with the operative room lights turned off, the area was stimulated with a 780 nm light beam, emitted by a LED (Light Emitting Diode) Thorlabs Model M780 L-C1 (Thorlabs GmbH, Dachau, Germany) with a bandpass excitation filter of 769 nm (+/− 41 nm) Edmund Optics 84,123 (Edmund Optics, Barringtion, NJ, USA), powered by a LED driver T-cube 1200 mA (Thorlabs GmbH, Dachau, Germany).

Simultaneously the stimulated area was visualized through an image acquisition system composed by a night vision goggle device iGen Nightviewer 20/20 nightOwl (Night Owl Optics, El Paso, Texas, USA) with a bandpass acquisition filter of 832 nm (+/− 28 nm).

In the last two cases, the acquisition system was connected to a Personal Computer through an USB acquisition board Video Grabber SVG 2.0 A3 Silvercrest (Targa GmbH Soest, Germany) using the Cyberlink PowerDirector 12 software (Cyberlink, New Taipei City, Taiwan), which allowed the recording of images and their analysis with Image J software (National Institutes of Health, Bethesda, Maryland, USA).

After visual identification of the parathyroid glands, confirmed with autofluorescence, the decision of which gland to excise was made by the surgeon based on the appearance and size of the identified glands.

The excised glands were immediately sent for frozen section examination. Venous blood samples were collected for Parathyroid hormone (PTH) determination at four different times: 1- after anaesthetic induction (baseline) 2- Pre-excision 3 – five minutes after excision 4 –ten minutes after excision.

The procedure was completed when Miami Criteria [14] were fulfilled. Determinations of serum calcium and PTH were done before hospital discharge (24 h after surgery) and 15 days after surgery in all patients.

In all five patients it was possible to identify the two autofluorescent parathyroid glands that had been previously visually identified (Table 2).

**Table 2:** Surgery data

Patient	Surgery	Pt identified/sought	Duration of surgery (minutes)	Pathological diagnosis
1	Excision of right inferior parathyroid	2/2	45	Oxyphilic cells adenoma
2	Excision of right superior parathyroid	2/2	50	Principal cells adenoma
3	Excision of right superior parathyroid Right thyroid lobectomy	2/2	65	Principal cells adenoma
4	Excision of right superior parathyroid Right thyroid lobectomy	2/2	45	Oxyphilic cells adenoma
5	Excision of right superior parathyroid	2/2	35	Principal cells adenoma

Abbreviation: *Pt* parathyroid glands

Operative times varied between 25 and 65 min (average 48 min).

**Fig. 1:**
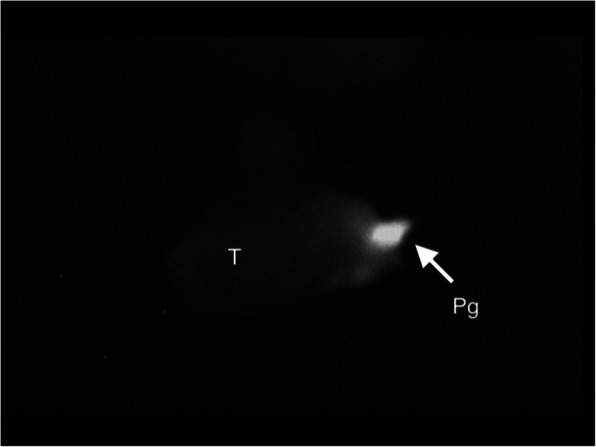
Autofluorescence of thyroid (T) and parathyroid (Pt) captured at exploration of the superior pole of thyroid (right side). We can see that thyroid gland also emits autofluorescence but with considerably less intensity than parathyroid

Quantification of fluorescence intensity by Image J software undertaken in the two last patients showed a marked difference between thyroid and parathyroid autofluorescence, with parathyroid intensity being almost 2 times higher (Fig. 1).

Obtained values of intensity (arbitrary units) and ratios are presented on Table 3.

**Table 3:** Quantification of autofluorescence with Image J software

	Patient 4	Patient 5
AF parathyroid adenoma	228	243
AF normal parathyroid	225	248
AF thyroid	110	125
Ratio AF adenoma/ Normal Pt	1.013	0.980
Ratio AF Adenoma/Thyroid	2.072	1.944
Ratio AF Normal Pt/ Thyroid	2.045	1.984

Abbreviations: *AF* autofluorescence, *Pt* parathyroid gland

Units: arbitrary units (imageJ software)

No statistical comparison was done due to the small sample size.

Visually there was no relevant difference between the perceived intensity of fluorescence of normal and pathological parathyroid glands.

Frozen section examination of the excised glands showed in all five cases the presence of a parathyroid adenoma, which was further confirmed by conventional pathological examination.

No parathyroid tissue was found within the lobectomy specimens.

Determinations of intra-operative PTH were performed in four patients (Table 4), showing a decrease of the PTH levels fulfilling Miami Criteria in use in Hospital dos SAMS [14].

**Table 4:** Calcium and PTH determinations

Patient	Pre-op Calcium	Pre-op PTH	Io PTH max	Io PTH min	Variation Io PTH	Calcium 24 h	PTH 24 h	Calcium 15 days	PTH 15 days
1	12	267	388	31.8	−90%	8.4	8.9	9.0	32
2	11.4	102	171	19	−88%	8.5	26	9.9	44
3	10.6	139	*	*	*	8.6	50	9.7	91
4	10.8	142	114	18.9	−83%	9.4	3	8.5	52
5	11.8	209	100	4	−96%	8.9	3	8.8	86

Abbreviations: *Pre-op* pre-operative, *PTH* parathyroid hormone, *io* intra-operative. *Intra-operative PTH not measured (failure of the equipment)

Units: Calcium: mg/dl, PTH: pg/ml

Normal values: Calcium 8,5–10,4 mg/dl PTH 11–65 pg/ml

In one patient these determinations were not performed due to technical problems with the analysis system. Results of calcium and PTH (pre-operative, intra-operative, 24 h and 15 days) are presented in Table 4.

During the six month follow-up period there were no cases of persistent hyperparathyroidism.

## Discussion and conclusions

Autofluorescence of parathyroid glands, when submitted to a near infrared light, allows their discrimination from other cervical tissues and may be useful in an operative setting.

Although the initial work of Paras et al. [5] had been directed to surgery of the thyroid gland, the autofluorescence of pathological parathyroid glands, namely adenomas and hyperplasia allows its utilization in surgery of hyperparathyroidism, helping in the localization of the glands [15].

The results of the surgery for primary hyperparathyroidism have always been highly dependent of surgeons’ experience [16].

As this surgery is less frequent than thyroid surgery, experience is not easily acquired outside reference centres. In addition, there are not many useful intra-operative tools for helping less experienced surgeons [17]. Furthermore, pre-operative localization tools are sometimes discordant, and in these cases, a bilateral neck exploration may be necessary to identify the affected glands [17].

In our series of five consecutive patients with non-concordant cervical ultrasonography and sestamibi scan, the utilization of the developed device facilitated the identification of the parathyroid glands although it could not differentiate normal from pathological glands, as there was no visually significant difference in fluorescence intensity. Nevertheless, the confidence of the surgeon in the identification of parathyroid glands was greatly improved with its use.

The small size of our sample doesn’t allow conclusions about the intensity of autofluorescence of normal and abnormal parathyroid glands, an issue that needs further evaluation.

Whereas the results of Falco et al. [9] showed higher intensity of autofluorescence of parathyroid adenomas than normal parathyroid glands, the results of Kose et al. [18] showed higher intensity of autofluorescence of normal glands. Ladurner et al. [7] and Squires et al. [19] didn’t find significant difference between normal and abnormal glands.

Larger studies with equivalent methodologies will be necessary to completely clarify the problem.

We also did not observe relevant differences in fluorescence intensity between adenomas with predominance of either principal cells or oxiphylic cells.

As the differences of fluorescence intensity between normal and pathological glands are non-significant, it seems unlikely that this measurement could be useful for the discrimination of affected glands.

The decision about which gland to excise remained dependent of the surgeons evaluation supported by frozen section examination and intraoperative PTH determinations.

However, this device could be useful for less experienced surgeons, with less visual training on identifying parathyroid glands.

As the current device used in this work is still in the process of improvement, it has some important limitations, namely the low image quality and the need to switch off the operative lights when in use.

Although near infrared light has some capacity of penetration into tissues (less than 5 mm), some dissection might still be necessary for parathyroid identification.

The ability of this technique for the identification of the rare true intrathyroid parathyroid glands remains uncertain [7].

Regardless of these limitations, our results suggest that the utilization of autofluorescence could be of great value for helping surgeons operating patients with primary hyperparathyroidism and even contribute to shorten the operative time.

The identification of parathyroid glands with autofluorescence could minimize the lesser experience of the surgeon performing parathyroid surgery, possibly reducing the need of a substantial visual training and improving the confidence of the surgeon and the speed of the procedure, namely in cases of non concordant ultrasonography and sestamibi scans, for which it is necessary to identify more than one gland.

Although these results need validation with studies enrolling a greater number of patients, the potential benefits in surgical practice, the ease of assemblage and use of our device, and the fact that it costs only a fraction of the devices recently launched in the market, may ultimately allow a more widespread use of this technique.

## Data Availability

All data generated or analyzed during this study are included in this published article.

## Abbreviations

LED: Light emitting Diode
PTH: Parathyroid hormone
